# Efficient genome editing using modified Cas9 proteins in zebrafish

**DOI:** 10.1242/bio.060401

**Published:** 2024-03-28

**Authors:** Laura Dorner, Benedikt Stratmann, Laura Bader, Marco Podobnik, Uwe Irion

**Affiliations:** Max Planck Institute for Biology, RG Colour Pattern Evolution, Tuebingen, Max-Planck-Ring 5, 72076 Tuebingen, Germany

**Keywords:** Zebrafish, *albino*, *kcnj13*, CRISPR, Pigment pattern evolution

## Abstract

The zebrafish (*Danio rerio*) is an important model organism for basic as well as applied bio-medical research. One main advantage is its genetic tractability, which was greatly enhanced by the introduction of the CRISPR/Cas method a decade ago. The generation of loss-of-function alleles via the production of small insertions or deletions in the coding sequences of genes with CRISPR/Cas systems is now routinely achieved with high efficiency. The method is based on the error prone repair of precisely targeted DNA double strand breaks by non-homologous end joining (NHEJ) in the cell nucleus. However, editing the genome with base pair precision, by homology-directed repair (HDR), is by far less efficient and therefore often requires large-scale screening of potential carriers by labour intensive genotyping. Here we confirm that the Cas9 protein variant SpRY, with relaxed PAM requirement, can be used to target some sites in the zebrafish genome. In addition, we demonstrate that the incorporation of an artificial nuclear localisation signal (aNLS) into the Cas9 protein variants not only enhances the efficiency of gene knockout but also the frequency of HDR, thereby facilitating the efficient modification of single base pairs in the genome. Our protocols provide a guide for a cost-effective generation of versatile and potent Cas9 protein variants and efficient gene editing in zebrafish.

## Teaser

**Summary:** We show that modifications of the Cas9 protein improve the genome editing versatility and efficiency of the CRISPR system in zebrafish, thus simplifying precise genetic modifications and allele swaps.

## INTRODUCTION

During the last decade the CRISPR/Cas system has greatly facilitated genome editing in many organisms, including so-called ‘model organisms’, like mouse, *Drosophila* or zebrafish (Housden et al., 2017). The system is based on an RNA-dependent endonuclease, very often the Cas9 protein from *Streptococcus pyogenes*, and an RNA molecule, the guide RNA (gRNA), which provides sequence specificity through base-pairing with the target DNA (Jinek et al., 2012). In addition to the base pairing of RNA and DNA a short motif, the proto-spacer adjacent motif (PAM), needs to be present for enzyme activity. In the case of Cas9 from *S. pyogenes* the PAM sequence is NGG (Jinek et al., 2012), which leads to some constraint when searching for potential target sites in the genome of interest. The generation of knockout alleles of protein-coding genes with the CRISPR/Cas system is most often achieved by introducing targeted DNA double-strand breaks (DSBs) *in vivo* and the subsequent cellular repair by non-homologous end-joining (NHEJ), which is error prone and frequently leads to small deletions or insertions (indels) disrupting the open reading frame (Hruscha et al., 2013). More precise genome editing can be achieved either by employing base editors (Pickar-Oliver and Gersbach, 2019) or by prime editing (Anzalone et al., 2019). Both methods work without the need to introduce DNA DSBs at the target site. Base editors rely on a fusion of Cas9 nickase with a single-stranded DNA deaminase enzyme and can catalyse the conversion of C:G base pairs to T:A (cytosine base editors), or A:T to G:C (adenine base editors) in a relatively small editing window. For prime editing Cas9 nickase is fused to a reverse transcriptase domain, which is used to introduce the gene edit via reverse transcription. An extended gRNA, the prime editing guide RNA (pegRNA), specifies not only the target site but also provides the primer binding sequence that hybridises with the 3′ end of the nicked DNA and the reverse transcription template containing the desired sequence change (Anzalone et al., 2020). A third and somewhat easier method to introduce DNA changes with base pair precision is based on the cellular homology-directed repair (HDR) pathway of DNA DSBs by providing a suitable repair template as donor. The repair templates might either be DNA fragments with long homology arms or single stranded oligonucleotides (Albadri et al., 2017). To overcome the targeting limitations of Cas9 an engineered variant that has a greatly relaxed PAM requirement has been generated (Walton et al., 2020). This protein, named SpRY, contains five point mutations compared to conventional Cas9 and was shown to possess near-PAMless endonuclease activity in cultured human cells (Walton et al., 2020). While SpRY allows for greater flexibility in the selection of target sites in a given genome, it might come with reduced stringency and therefore a greater chance of off-target activity, which needs to be considered. The protein has been used in zebrafish to induce loss-of-function mutations by indels (Liang et al., 2022), or in combination with base editors to produce precise genome edits (Liang et al., 2022; Rosello et al., 2022). In both cases the efficiency of the genome edit was variable and appeared to be highly locus specific. A co-selection method with a visible marker gene knockout was employed by Rosello et al. to increase the rate of identifying edited larvae (Rosello et al., 2022). In eukaryotes the Cas9 protein needs to be targeted to the nucleus in order to be active. This is frequently achieved by tagging the protein with a nuclear localization signal (NLS) derived from the SV40 large T-antigen. However, it was shown that in medaka (*Oryzias latipes*) embryos the SV40 NLS does not direct efficient nuclear import during very early stages of development (Inoue et al., 2016), which is when Cas9 activity would be most effective to generate low levels of mosaicism and high chances of germ line transmission of the engineered allele. An optimized artificial NLS (aNLS) was found that leads to prominent nuclear localization of GFP immediately after fertilization in medaka (Inoue et al., 2016). In zebrafish (*Danio rerio*) the inclusion of this aNLS sequence in combination with a myc-tag and a flexible linker into a high efficiency-tag (hei-tag) was shown to boost genome editing efficiency of Cas9 protein and of cytosine-to-thymine base editors (Thumberger et al., 2022). Here, we confirm that the SpRY variant of Cas9 can be used for efficient genome editing in zebrafish. However, we also find that not all tested sites are targeted with the same efficiency and that the individual target sequence, independent of the potential PAM sequence, might be of great importance, which means that efficiencies need to be assessed on a one-by-one basis. We also show that the addition of an aNLS to the Cas9 or SpRY proteins enhances their efficiency to a degree that allows the precise exchange of one codon in the genome of zebrafish without the need for a visible read-out or pre-selection of the F_0_ larvae. We use the improved method to precisely change the coding sequence for *kcnj13* in zebrafish into the sequence found in *Danio aesculapii*, its closest sister species (Podobnik et al., 2020, 2023), which had previously failed following published protocols for HDR. Zebrafish carrying the edited sequence do not show any visible phenotype, thus demonstrating that the Kcnj13 proteins from both species are functionally equivalent. In summary, we describe a very simple and robust method for precise genome editing that makes use of purified proteins and *in vitro* transcribed RNAs, and thus has the potential to be used in many laboratories and strengthen zebrafish as a model organism for bio-medical research.

## RESULTS

### Efficient *albino* knockout with the SpRY protein

**Fig. 1.**
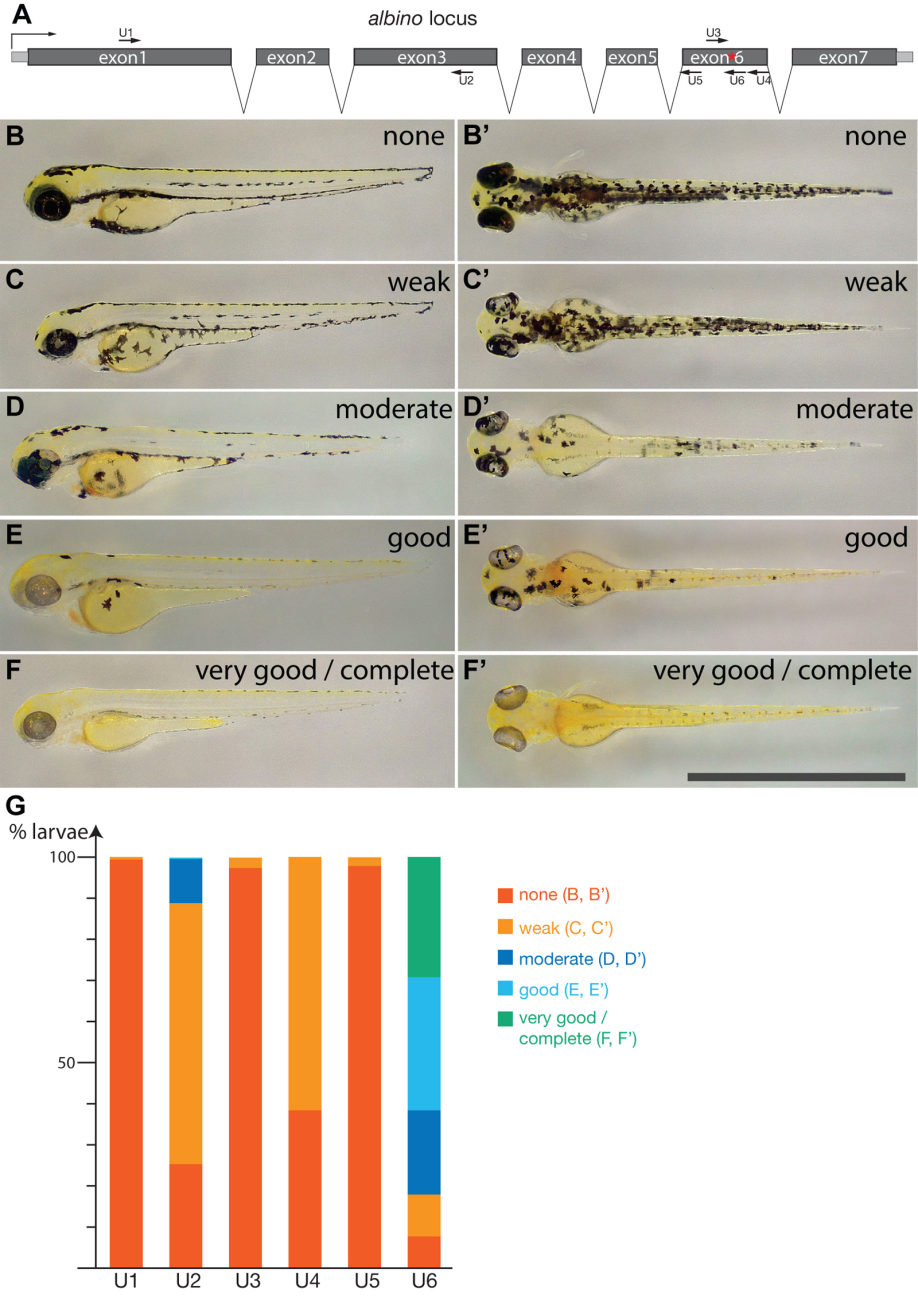
**Efficient knockout of the *albino* gene using the SpRY protein.** In A the *albino* locus is schematically shown, exons are in grey, introns are shown as gaps, not to scale. The coding sequence is in dark grey and the different target sites in exons 1, 3 and 6 are indicated, the position of the *alb^b4^* mutation in exon 6 is marked by a red asterisk. The different categories for the evaluation of the knockout efficiency are shown in B-F′. Lateral views (B-F) and dorsal views (B′-F′) of larvae 3 dpf are shown, scale bar: 2 mm. Category 1, no knockout (none) (B), (B′), category 2 weak (C), (C′), category 3 moderate (D), (D′), category 4 good (E), (E′) and category 5 very good/complete knockout (F), (F′). In G the results for the six target sites tested are shown. For detailed results see Table S1.

To assess whether a modified version of the Cas9 protein (SpRY), which has been reported to allow near-PAMless genome targeting in cultured human cells (Walton et al., 2020), is also active in zebrafish we used the *albino* (*alb/slc45a2*) gene as target. Mutations in *alb* can be generated with very high efficiency using the conventional Cas9 protein, and it is possible to repair a premature stop codon in the *alb^b4^* mutant by co-injection of a donor-plasmid for homology-directed repair (Irion et al., 2014). We generated six single guide RNAs (sgRNAs) to target different positions in three exons of *alb* (Fig. 1A). The target sites were selected to have GC contents of 50-65% and different PAMs, which were among the originally identified most efficient sequences (Walton et al., 2020) (see Table 1). The corresponding sgRNAs were injected together with a purified fusion protein of SpRY C-terminally tagged with mCherry, and also containing N-terminal Strep- and His-tags for purification and an SV40 large T NLS (for simplicity referred to a SpRY in the rest of the manuscript), into TU embryos. For the evaluations of these *alb* knockout experiments we examined the mosaic F_0_ larvae after 3 days and classified them into five categories according to the severity of the *alb* phenotype (Fig. 1B-F’): 1 (none), no loss of pigmentation discernible; 2 (weak), loss of pigmentation in individual cells, usually only clearly visible in the retinal pigment epithelium; 3 (moderate), regional loss of pigmentation clearly visible; 4 (good), maximum of 50 pigmented cells left; 5 (very good/complete), maximum of five pigmented cells. For three of the tested target sites (U1, U3, U5) we found no or only very weak activity of the injected SpRY-RNP. In two cases (U2 and U4) we found moderate activity, but in one case (U6) the activity was high, with 49% to 82% (61.6% average) of the larvae falling into categories 4 and 5 (Fig. 1G; Table S1). This is comparable to the knockout activity we regularly achieve with unmodified Cas9 protein.

**Table 1.**
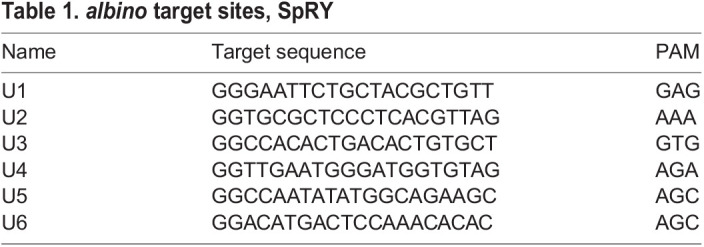
*albino* target sites, SpRY

### Repair of the *albino^b4^* mutation with the SpRY protein and oligonucleotide donors

**Fig. 2.**
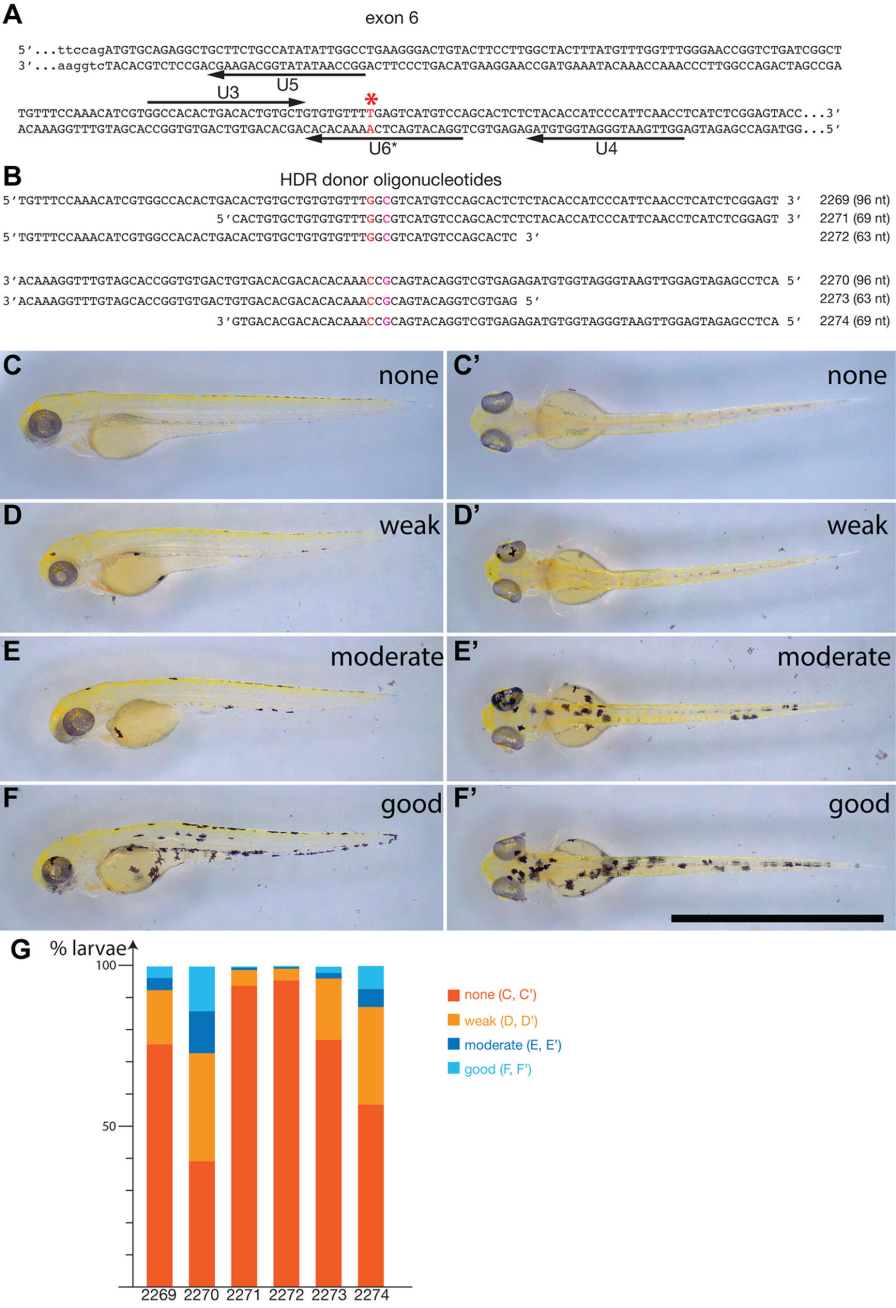
**Homology directed repair of the *albino^b4^* mutation using the Cas9 variant SpRY.** In A the partial sequence of exon 6 of the *albino* gene is depicted, coding sequence in capital letters, intron sequence in small letters. The target sites U3, U4, U5 and U6* are indicated by arrows; the mutation in *alb^b4^* leading to a premature stop codon is shown in red and marked with an asterisk. In B the oligonucleotides used as donors for HDR are shown. The two base pairs that are altered are shown in red; note the 5′ and 3′ ends of the oligonucleotides indicating the DNA strand they correspond to. The different categories for the evaluation of the repair efficiency are shown in C-F′. Lateral views (C-F) and dorsal views (C′-F′) of larvae 3 dpf are shown, scale bar: 2 mm. Category 1, no repair (none) (C), (C′), category 2 weak (D), (D′), category 3 moderate (E), (E′) and category 4 good repair (F), (F′). In G the results for the six tested donor oligonucleotides are shown. For detailed results see Table S2.

The target site for the U6 sgRNA overlaps with the mutation in *alb^b4^*, a premature stop codon in exon 6 (Fig. 2A) (Dooley et al., 2013), thus, making it possible to use it in a repair experiment. We adjusted the sgRNA accordingly (U6*) to target this site in *alb^b4^* mutants. In control experiments, when injected into TU wild-type fish, we found that one mis-match in the target sequence is enough to completely abolish the endonuclease activity of the SpRY protein. We then co-injected SpRY and the sgRNA U6* in *alb^b4^* mutants together with oligonucleotides as donor templates for homology-directed repair of the mutation (Fig. 2B). To evaluate these repair experiments we grouped the larvae into four categories: 1 (none), no pigmented cells visible; 2 (weak), fewer than 15 pigmented cells; 3 (moderate), 15-25 pigmented cells; 4 (good), more than 25 pigmented cells (Fig. 2C-F′). Using oligonucleotides of different lengths, we found that one of them (TÜ2270) leads to very high numbers (up to 66%) of larvae with pigmented cells, indicative of the repair of the *alb^b4^* mutation (Fig. 2G). This donor oligonucleotide corresponds to the DNA sense-strand with regard to the sgRNA molecule. Other oligonucleotides, either shorter in length or corresponding to the antisense DNA strand, lead to lower HDR efficiencies (Fig. 2G, Table 2). However, most of the larvae we obtained in this experiment showed only very few pigmented cells and thus fell into categories 2 and 3, much fewer (max. 16%) larvae fell into category 4 with more than 25 pigmented cells (Fig. 2G). When we raised these fish of category 4 to adulthood and crossed them to *alb^b4^* mutants we found that only one fish out of 16 showed germ-line transmission of the repaired allele.

**Table 2.**
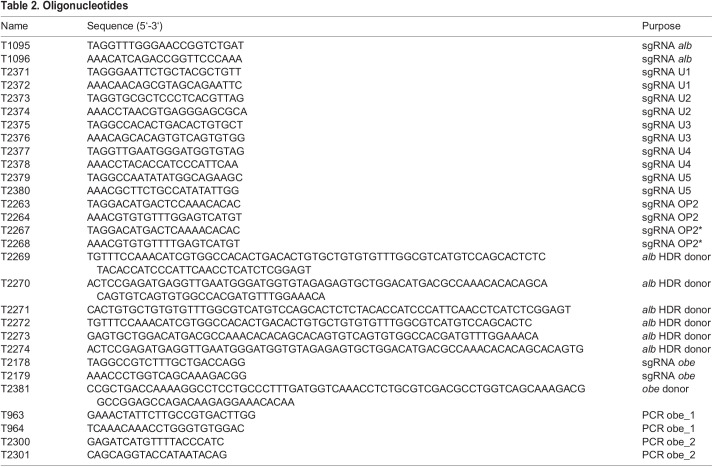
Oligonucleotides

### Enhanced efficiency by addition of an aNLS

**Fig. 3.**
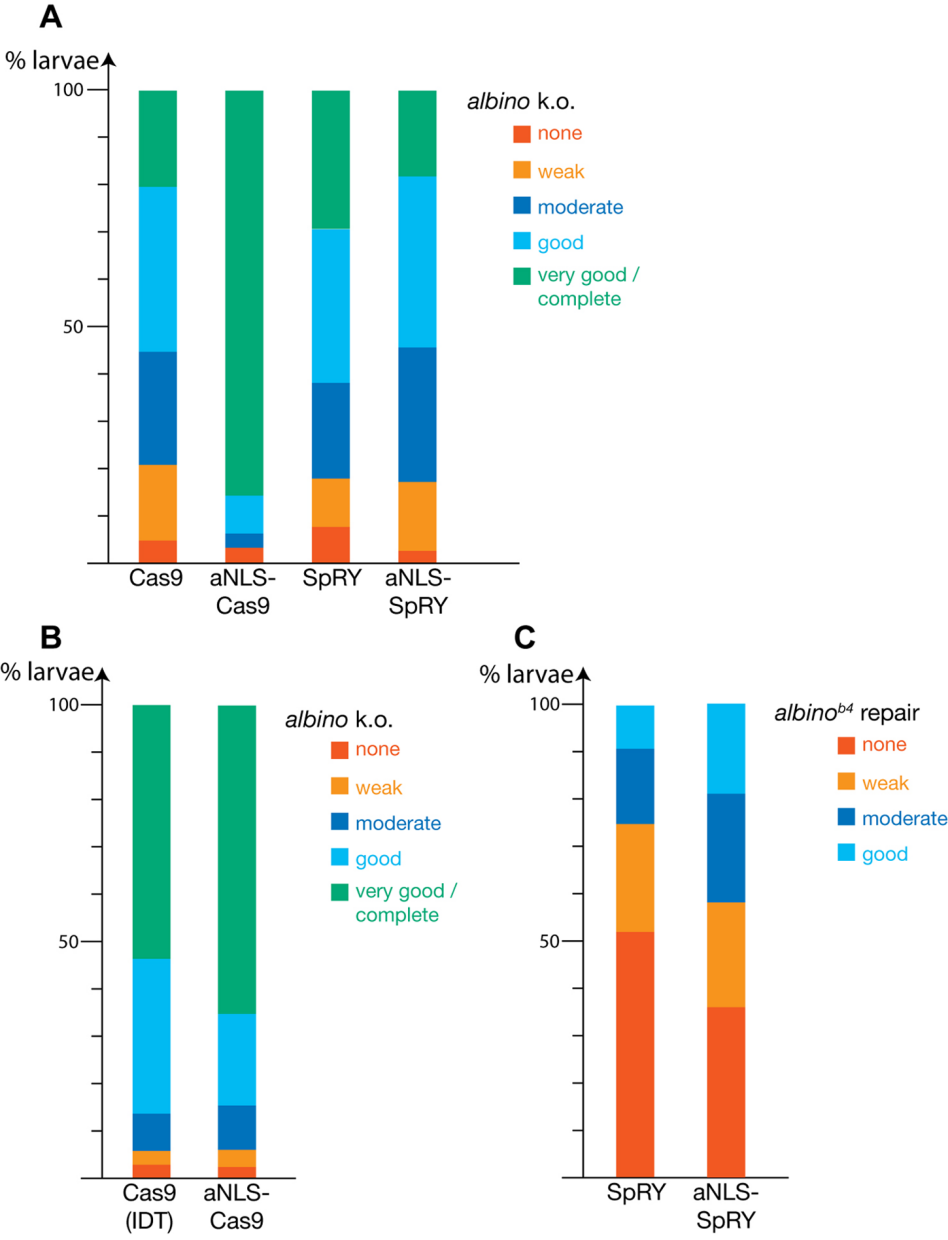
**Improvement of knockout and repair efficiencies by adding an aNLS.** In A the efficiencies of knocking out the function of the *albino* gene are compared between Cas9 and aNLS-Cas9 and SpRY and aNLS-SpRY. Whereas in the case of Cas9 the addition of an aNLS leads to a significantly higher efficiency, 93.5% in the best two categories compared to 55.1%, this is not the case for SpRY. Detailed results see Table S3. Purified aNLS-Cas9 performs as well as commercially available Cas9 protein (Alt-R™ S.p. Cas9 V3, IDT) when tested for the knockout of the *albino* gene (B). In addition, the repair efficiency for aNLS-SpRY is significantly higher compared to SpRY, with 19% versus 9.1% in the category ‘good’ (C). For detailed results see Tables S4 and S5.

We hypothesized that the low efficiency of HDR, despite the relatively high frequency (up to 66% of F_0_ larvae with some pigmented cells), might be due to inefficient nuclear import of the Cas9 protein during early stages of embryonic development. For medaka it was shown that an aNLS (sequence: PPPKRPRLD) improves nuclear import of proteins during early stages of development compared to the most commonly used SV40-NLS (sequence: PKKKRKV) (Inoue et al., 2016). In zebrafish the inclusion of the aNLS in combination with a myc-tag and a flexible linker into a so-called hei-tag increased the overall genome editing efficiency of different Cas9 protein variants in combination with a reduction in allele variance, indicating enhanced activity during early developmental stages (Thumberger et al., 2022). We added the aNLS sequence to the N- and C-termini of mCherry tagged Cas9 and SpRY proteins and compared the activity of the resulting fusion proteins (aNLS-Cas9 and aNLS-SpRY) with the ‘normal’ Cas9 or SpRY, which both carry an SV40NLS C-terminally. We found that addition of the aNLS sequence leads to a very obvious increase in the *alb* knockout efficiency when using the Cas9 protein, with more than 85% of the injected F_0_ larvae showing a complete *alb* phenotype with no melanophore pigmentation (Fig. 3A; Table S3). However, in the case of the SpRY protein the addition of the aNLS did not result in an improvement of the knockout efficiency (Fig. 3A).

Nevertheless, using the aNLS-SpRY protein resulted in a substantial increase in the efficiency for HDR-mediated repair of the *alb^b4^* mutation. We found that the number of larvae in category 4, with more than 25 pigmented cells, more than doubled from 9.1% to 19.8% (Fig. 3C; Table S4).

### Comparison with commercial Cas9 protein

To compare our own purified aNLS-Cas9 protein with a commercially available protein (Alt-R™ S.p. Cas9 V3, IDT) we injected them in parallel with *alb* sgRNA into TU embryos to generate *alb* knockouts. We found that when using 500 ng/µl of the protein, knockout mutations in *alb* were induced with comparable very high frequencies in both cases, with more than 50% of the larvae falling into category 5 (Fig. 3B; Table S5). At concentrations of 1000 ng/µl mortality rates for our own protein were too high to allow meaningful comparisons.

### Allele exchange by HDR at the *kcnj13* locus

**Fig. 4.**
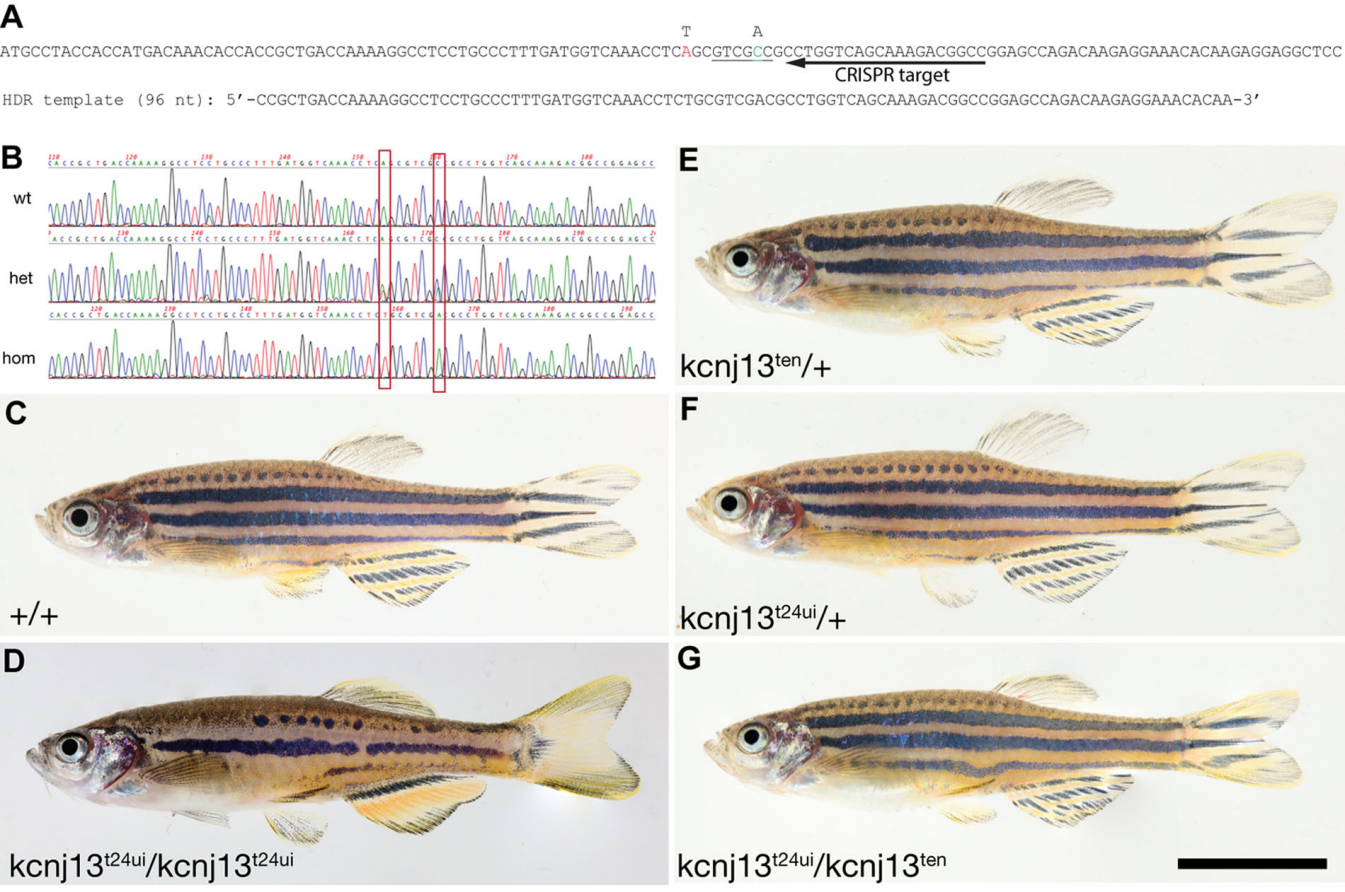
**Allele exchange in the *obelix* gene.** In A the start of the coding sequence for *obe* is shown. The CRISPR target site is indicated by an arrow, the two point mutations introduced by HDR are shown in red and green, respectively, the recognition site for SalI that is generated by the HDR is underlined. The oligonucleotide used as donor DNA is also depicted. In B sequences from wild-type fish, and fish heterozygous or homozygous for the engineered allele are shown, the two positions that were engineered are boxed. There are no discernible differences between wild-type siblings (C) and fish heterozygous for the engineered allele (*kcnj13^ten^*/+) (E), nor between fish that carry a *kcnj13* loss-of-function allele over a wild-type allele (*kcnj13^t24ui^*/+) (F) or over the engineered allele (*kcnj13^t24ui^*/ *kcnj13^ten^*) (G). For comparison, a fish homozygous mutant for a loss-of-function allele of *obe* (*kcnj13^t24ui^*/*kcnj13^t24ui^*) is shown in D, scale bar: 1 cm.

Finally, we employed the HDR method for the directed exchange of one specific base pair in the genome of zebrafish. We used the method do change one codon in the *obelix* (*obe/kcnj13*) gene, which is required for the establishment of the striped pigmentation pattern in adult zebrafish (Haffter et al., 1996). Mutants homozygous for a loss-of-function allele of *obe* show a pattern of fewer and disrupted dark stripes on the flanks and in the anal and tailfins (Podobnik et al., 2020) (Fig. 4D). In *Danio aesculapii*, the closest sister species to zebrafish, the homologous gene is also required for patterning, although the pattern is very different, consisting of vertical bars. Only one non-synonymous site in *kcnj13* is fixed between the two species, with CAG (Gln23) in zebrafish compared to CTG (Leu23) in *D. aesculapii* (Podobnik et al., 2020, 2023). Therefore, we targeted the first coding exon of *obelix* (*obe*/*kcnj13*) and co-injected donor-oligonucleotides to change this codon from CAG to CTG. As we have no visible read out in this case we designed the donor-oligonucleotides to contain a second base change that will not affect the coding potential but introduce a novel target site for the restriction enzyme SalI (Fig. 4A). We screened some of the injected F_0_ larvae by PCR and restriction enzyme digest to make sure that the desired HDR outcome was present. While Cas9 protein without aNLS did not yield any positive results, in the case of aNLS-Cas9 we could detect the presence of a SalI restriction site in six PCR-fragments from 16 larvae tested, when the donor-oligonucleotide corresponded to the DNA sense strand. Sequencing of the cloned PCR-products confirmed that the allele exchange had taken place. However, we found that other changes near the target site had also occurred rather frequently and just one out of four cloned DNA fragments had only the desired changes. We raised approximately 100 of the injected F_0_ larvae to adulthood and then tested sperm from 64 adult male fish for the presence of the engineered allele in the germ cells. This led to the identification of eight candidate fish. When outcrossed to wild-type females we identified the presence of the SalI site in F_1_ offspring of six of these candidate fish. Sequencing showed that in five cases additional mutations were present, either point mutations probably caused by imperfect repair templates or larger deletions most likely introduced during cellular DNA damage repair. However, in the offspring of one founder only the intended DNA changes were present and no further mutations detectable. The heterozygous F1 fish, which carry the precisely engineered allele, are indistinguishable from their wild-type siblings (Fig. 4C,E). In addition, we crossed the identified founder fish to a *kcnj13* mutant female and identified trans-heterozygous individuals in the resulting offspring. These fish, carrying a *kcnj13* loss-of-function allele and the engineered *kcnj13* allele, also show a wild-type phenotype and are indistinguishable from heterozygous siblings (Fig. 4F,G). These results show that the proteins from both species can perform the same functions in zebrafish.

## DISCUSSION

Genome engineering with the CRISPR/Cas9 system in zebrafish is very robust and can be used by any researcher with only minimal prior training (Uribe-Salazar et al., 2022). This makes the method ideal for teaching purposes, especially when combined with an assay that allows visible quantification of knockout efficiencies after only 2 or 3 days, as is the case for the *alb* gene. While we found some variability in our results (see for example Table S3), we could reproduce all of them in several independent experiments. The observed variability most likely depends on a combination of different factors, such as the purity of the CRISPR components, mainly the different sgRNAs and oligonucleotides used, which were often different between experiments, whereas the proteins were purified once and stored as frozen aliquots. In addition, with more training the experimenters generally get better at performing injections and a higher proportion of the injected embryos survive. Our purified aNLS-Cas9 protein performed marginally better than a commercially available Cas9 (Alt-R™ S.p. Cas9 V3, IDT) when injected at our standard protein concentration of 500 ng/µl, which corresponds to 3.1 µM for Alt-R™ Cas9, a slightly lower concentration than the 4 µM recommended by the company. At the higher concentration of 1000 ng/µl, mortality rates were very high for the embryos injected with our own purified aNLS-Cas9 making a meaningful comparison impossible. A further purification step, for example by gel filtration, might improve the purity of the protein and lead to lower mortality at high concentrations.

A number of different CRISPR-based methods exist for precise base pair changes in eukaryotic genomes (Anzalone et al., 2020). Besides HDR, two alternative methods are prime editing (Anzalone et al., 2019; Porto et al., 2020) and the use of base editors (Porto et al., 2020). While prime editing allows the introduction of a variety of different genomic changes, it requires the careful design and synthesis of relatively long pegRNAs and the selection of the appropriate prime editor protein (Doman et al., 2023), which makes the method technically more challenging than HDR. Base editors, on the other hand, are active in just a small editing window, thus limiting the accessible target sites. A constraint that might be overcome by the use of near PAM-less editors (Rosello et al., 2022) or the *de novo* generation of the desired PAM in the genome (Pakari et al., 2023), which is again more laborious than genome editing via HDR. But, most importantly, base editors only produce certain base changes, either C to T for cytosine base editors, or A to G for adenine base editors. This would not allow the changes we wanted to introduce into the *alb* or *kcnj13* genes, T to G and T to A, respectively. Therefore, HDR is still a very convenient method for precise base changes in the zebrafish genome, especially when synthetic oligonucleotides can be co-injected as donor templates (Carrington et al., 2022). It is, however, desirable to find a target site for the endonuclease as close as possible to the position that should be changed. To test if Cas9 variants with reduced PAM-specificity can be used in zebrafish as a possibility to expand the target range of the CRISPR system, we tested SpRY. This protein is a variant of Cas9 that was engineered to have a less stringent requirement for the PAM sequence and was found to be nearly PAM-less (Walton et al., 2020). While the relaxed stringency due to the reduced requirement for the PAM sequence of SpRY might lead to higher off-target activity, we did not find any higher rates of lethality or malformations in the injected embryos. For two of the target sites, we tested in the *alb* gene we found that the activity of SpRY was rather weak, for three additional sites it was almost undetectable. However, for the sixth target site the knockout efficiency was very high, comparable to the high efficiency we regularly obtain using normal Cas9 protein (Irion et al., 2014) (see Fig. 1). This is similar to previous reports, which also found varying efficiencies for SpRY in generating indels or for base editing with several target sites being apparently inaccessible (Liang et al., 2022; Rosello et al., 2022). A direct comparison of our results with these previous reports is difficult, however, because of the different methods employed to measure the editing efficiency. Whereas we relied on a bi-allelic knockout of the target gene in one specific larval cell type, the melanophores, to assess gene targeting efficiency these other studies used direct sequence analysis of the target regions. While sequencing is clearly a more sensitive method as it does neither require a bi-allelic knockout nor is it restricted to one specific cell type, our approach is still valid as it is pragmatic and low cost. This is because in most experiments either the F_0_ phenotype of the induced mutation will be assessed or germ line transmission is required, and in both cases high efficiencies are crucial. While we cannot state what underlies the differences in efficiency for the different target sites in the *alb* gene, we realise that while two of the target sites, U5 and U6, share the same PAM sequence and GC content the editing efficiencies are very different (see Table 1 and Fig. 1G). This finding is slightly different from a previously published analysis, which identified some PAM dependence of SpRY targeting efficiencies in zebrafish in addition to locus specific effects (Liang et al., 2022). A possible explanation for this discrepancy could be the different read outs used to assess targeting efficiency, as mentioned above. In conclusion, we find that SpRY might be a good option to expand the targeting range of the CRISPR/Cas system in zebrafish, but potential target sites need to be evaluated individually.

In our initial experiments using SpRY to repair the premature stop codon in the *alb^b4^* mutant via HDR with oligonucleotides as donor DNA we found that the majority of all larvae only had very few pigmented melanophores. This indicates that the repair happens only after a lag phase of probably several hours after injection, otherwise we would expect larger clones of pigmented cells with the edited DNA. The reason for this might be the inefficient transport of the RNP into the nucleus during very early zygotic development, as has been shown to be the case in medaka for proteins carrying the SV40-NLS (Inoue et al., 2016). Indeed, the addition of an aNLS to the Cas9 protein markedly increased the number of injected larvae that showed a complete *alb* phenotype to over 85% in some of our experiments, with the average being over 65%. Similarly, the repair efficiency was also higher when using aNLS-SpRY (19.0%) compared to normal SpRY (9.1%). Whether the addition of a myc-tag and a flexible linker, as is required in the hei-tag (Thumberger et al., 2022), would increase the efficiency further still needs to be tested. The marked increase in efficiency we found using aNLS-Cas9 prompted us to use it for gene editing a position in the zebrafish *kcnj13* gene where a directly visible phenotypic read-out in larvae does not exist and where we did not succeed previously with the precise edit using a method that works efficiently for other loci (Irion et al., 2014). We employed the improved method to produce a precise change in the coding sequence of *kcnj13* in zebrafish. The gene is involved in the generation of the distinct pigmentation patterns in the two sister species zebrafish and *D. aesculapii*. We could previously show that the gene function has diverged between the two species (Podobnik et al., 2020) and that expression levels in hybrids differ depending on the parental origin of the allele, strongly suggesting that the evolutionary divergence is based on cis-regulatory changes (Podobnik et al., 2023). However, to rule out that the one non-synonymous change in the coding sequence of *kcnj13* that is fixed between the two species affects the function of the protein and might also contribute to the evolutionary divergence we introduced a point mutation in zebrafish that leads to an exchange of Gln^23^ to Leu, thus making it identical to the wild-type *kcnj13* allele from *D. aesculapii*. This mutation does not lead to any visible pigmentation defect, neither when homozygous nor when trans-heterozygous with a loss-of-function allele of *kcnj13* (see Fig. 4), thus confirming that the proteins from both species function identically during pigment pattern formation in zebrafish.

In summary, we show that the versatility of the CRISPR-system can be extended by the use of the SpRY protein variant and that the inclusion of an artificial NLS enhances the efficiency for the production of knockouts as well as knockins via HDR. Our improvements to the HDR method are easy to implement in different laboratory settings, the aNLS-tagged proteins, which can be expressed in bacteria and purified by standard methods using a His-tag and Strep-Tag in the same way as conventional Cas9 (Podobnik et al., 2023), can be used to simply substitute for regular Cas9. The highly efficient generation of *alb* knockouts makes it also possible to use it for teaching undergraduate students in courses.

## MATERIALS AND METHODS

### Fish husbandry and micro-injections

Zebrafish were bred and maintained as described previously (Brand et al., 2002). *D. rerio* wild-type Tuebingen (TU), *albino^b4^* (Chakrabarti et al., 1983), and *kcnj13^t24uI^* (Podobnik et al., 2020) strains were used. All animal experiments were carried out according to the local guidelines and approved by the Regierungspräsidium Tübingen.

The sgRNAs for the experiments were produced as described previously (Irion et al., 2014); for the oligonucleotides used see Table 2. The standard mix for micro-injections contained 500 ng/µl Cas9 or SpRY protein, 35 ng/µl *in vitro* transcribed sgRNA (MegaScript T7 kit, Invitrogen), 30 ng/µl single-stranded DNA oligonucleotides (for repair experiments) and 0.05% Phenol Red in PBS+300 mM NaCl, 150 mM KCl. One-cell stage embryos were injected with approx. 2 nl of this mix using a pneumatic system (pneumatic pico pump, WPI) and glass capillaries (Zebrafish-injection-pipettes blunt, BioMedical Instruments). Scoring of the *albino* phenotype was done at 3 days-post-fertilization (dpf).

### PCR and genotyping

For genotyping individual larvae at 1 dpf they were put in 100 µl 50 mM NaOH and incubated at 95°C for 30 min. Afterwards 10 µl of Tris-HCl pH 8 was added, and 1 µl was used as template for the subsequent PCR amplification with the DreamTaq Hot Start DNA Polymerase (Thermo Fisher Scientific).

To screen male F_0_ fish for potential germ-line transmission of the edited allele, sperm was extracted from anaesthetized fish by applying gentle pressure and collected with a glass capillary. The sperm sample was then treated identical to the larvae.

To achieve reliable amplification of the region of interest from the *obe* coding sequence a nested PCR was done. In the first round primers T963 and T964 were used, in a second round 1 µl of the first PCR was used as template with primers T2300 and T2301. For both rounds the following PCR conditions were used: Step 1: 95°C for 2 min, Step 2: 95°C for 20 s, Step 3: 62°C (−0.4°C per cycle) for 20 s, Step 4: 72°C for 30 s, repeat step 2 ten times, Step 5: 95°C for 20 s, Step 6: 58°C for 20 s, Step 7: 72°C for 30 s, repeat step 5 35 times, Step 8: 72°C for 3 min, Step 9: 8°C forever.

The PCR products were analysed by agarose gel electrophoresis, restriction digest with FastDigest SalI (Thermo Fisher Scientific) and Sanger sequencing (Genewiz, Azenta Life Sciences).

### Protein expression and purification

The different Cas9 proteins were expressed as fusions with C-terminally attached fluorescent proteins (mCherry or mVenus) in *E. coli* BL21(DE3)pLysS and purified using a double tag, 6xHis and twin-StrepTag. Briefly, the bacteria were transformed with one of the plasmids, NLS-Cas9-mCherry (GenBank: OP243709), NLS-SpRY-Venus (GenBank: OR801322), aNLS-Cas9-mCherry (GenBank: OR801323), or aNLS-SpRY (GenBank: OR801324), and grown overnight at 37°C on LB plates containing 50 µg/ml kanamycin. The bacterial colonies were scraped of the plates the next day, resuspended in 50 ml of LB medium, and used to inoculate 400 ml LB+kanamycin (starting OD_600_ of 0.1–0.2). The cultures were grown at 37°C with vigorous shaking until the OD600 reached 0.6 and then transferred to 18°C for 1 h. Protein expression was induced by the addition of IPTG (final concentration 0.5 mM), and the bacteria were grown for 18 h at 18°C and then harvested by centrifugation. The bacterial pellet was resuspended in 50 ml lysis buffer (20 mM Na-phosphate, 20 mM Tris-HCl, 600 mM NaCl, 150 mM KCl, 0.5 mM TCEP, 0.1% Tween20, pH=8) and the bacteria were gently lysed by freezing and thawing five times, then adding lysozyme to a final concentration of approx. 0.2 mg/ml and incubation on ice for 1 h. After centrifugation (4°C for 1 h at 3220 ***g***) the supernatant was filtered (1.2 µm filter) and then incubated for 2 h on ice on a rocking platform with 1 ml Protino Ni-NTA Agarose (MN, Macherey-Nagel GmbH, Düren, Germany) pre-equilibrated with lysis buffer. The agarose with the bound protein was recovered by centrifugation (4°C for 5 min at 900 ***g***), washed twice with 20 ml wash buffer 1 (= lysis buffer+20 mM imidazole) and then at room temperature packed into a 10 ml Polyprep Chromatography Column (Bio-Rad Laboratories, Feldkirchen, Germany). The protein was eluted from the column with 5 ml elution buffer 1 (= lysis buffer+500 mM imidazole). This eluate was applied on a second column, 1 ml StrepTactin (IBA Lifesciences, Göttingen, Germany) pre-equilibrated with lysis buffer, by gravity flow. The column was washed with 20 ml wash buffer 2 (= IBA Buffer W+300 mM NaCl) and the protein eluted with 1 ml elution buffer 2 (= IBA Buffer E+300 mM NaCl). The eluted protein was the dialyzed (SnakeSkin Dialysis Tubing, MWCO 10,000, Thermo Fisher Scientific) overnight using 3×2 l of PBS+300 mM NaCl and 150 mM KCl. The protein concentration was adjusted to 1 (or 2) mg/ml and 10 µl aliquots were frozen at −70°C.

For comparisons Cas9 protein [Alt-R™ S.p. Cas9 V3, glycerol-free, 10 mg/ml (62 µM)] was purchased from Integrated DNA Technologies, Leuven, Belgium.

## Supplementary Material

10.1242/biolopen.060401_sup1 Supplementary information
