# Supplementary material for: Efficient genome editing using modified Cas9 proteins in zebrafish
Source: Biol Open. 2024 Mar 28;13(4):bio060401. doi: 10.1242/bio.060401 (PMC10997048; doi:10.1242/bio.060401)
Supplement: Supplementary information [file biolopen-13-060401-s1.pdf]

Table S1. ***albino*** **k.o.; SpRY protein, different sgRNAs**

	none	weak	moderate	good	very good / complete
**U1**	165	5	0	0	0
	174	0	0	0	0
	163	0	0	0	0
	159	1	0	0	0
	3	0	0	0	0
	106	0	0	0	0
	243	0	0	0	0
**sum**	1013	6	0	0	0
%	99.4	0.6	0	0	0
N=1019					
**U2**	17	33	2	0	0
	20	32	2	0	0
	17	113	3	0	0
	24	108	11	0	0
	13	25	2	0	0
	29	27	3	0	0
	44	74	48	2	0
**sum**	164	412	71	2	0
%	25.3	63.5	10.9	0.3	0
N=649					
**U3**	197	19	0	0	0
	162	0	0	0	0
	157	0	0	0	0
	153	2	0	0	0
	62	0	0	0	0
	0	0	0	0	0
	75	0	0	0	0
**sum**	806	21	0	0	0
%	97.5	2.5	0	0	0
N=827					
**U4**	49	188	0	0	0
	102	134	0	0	0
	31	109	0	0	0
	42	101	0	0	0
	27	0	0	0	0
	23	13	0	0	0
	71	8	0	0	0

Biology Open • Supplementary information

**sum**	345	553	0	0	0
%	38.4	61.6	0	0	0
N=898					
**U5**	137	13	0	0	0
	152	0	0	0	0
	108	0	0	0	0
	107	1	0	0	0
	46	0	0	0	0
	36	0	0	0	0
	68	0	0	0	0
**sum**	654	14	0	0	0
%	97.9	2.1	0	0	0
N=668					
**U6**	0	1	14	20	20
	0	0	19	16	18
	1	5	4	16	2
	5	8	15	58	77
	43	51	79	97	69
**sum**	49	65	131	207	186
%	7.7	10.2	20.5	32.4	29.2
N=638					

Biology Open • Supplementary information

Table S2. ***albino*** **repair; SpRY protein, OP2* sgRNA, different donor oligonucleotides**

	none	weak	moderate	good	very good / complete
**TÜ2269**	65	15	3	8	
	69	5	7	3	
	45	15	2		
	98	27	2	2	
sum	277	62	14	13	
%	75.7	16.9	3.8	3.6	
N=366					
**TÜ2270**	56	36	12	20	
	23	24	11	9	
	25	90	35	37	
sum	104	90	35	37	
%	39.1	33.8	13.2	13.9	
N=266					
**TÜ2271**	71	2		1	
	301	18	3		
sum	372	20	3	1	
%	93.9	5.1	0.8	0.3	
N=396					
**TÜ2272**	27	7	1	1	
	295	6	1		
sum	322	13	2	1	
%	95.3	3.8	0.6	0.3	
N=338					
**TÜ2273**	64	12	2	1	
	141	39	3	4	
sum	205	51	5	5	
%	77.1	19.2	1.9	1.9	
N=266					
**TÜ2274**	50	22	4	8	
	141	81	15	16	
sum	191	103	19	24	
%	56.7	30.6	5.6	7.1	
N=337					

Table S3. ***albino*** **k.o.; Cas9 and SpRY proteins**

	none	weak	moderate	good	very good / complete
**Cas9**	1	9	6	15	9
	1	0	4	4	6
	5	7	11	11	6
	2	14	24	35	17
**sum**	9	30	45	65	38
%	4.8	16.0	24.1	34.8	20.3
N=187					
**aNLS-Cas9**	0	0	5	6	65
	3	0		7	58
	4	0	1	3	48
**sum**	7	0	6	16	171
%	3.5	0	3.0	8.0	85.5
N=200					
**SpRY**	0	1	14	20	20
	0	0	19	16	18
	1	5	4	16	2
	5	8	15	58	77
	43	51	79	97	69
**sum**	49	65	131	207	186
%	7.7	10.2	20.5	32.4	29.2
N=638					
**aNLS-SpRY**	4	11	10	23	19
	0	0	1	8	9
	4	36	80	85	30
**sum**	8	47	91	116	58
%	2.5	14.7	28.4	36.3	18.1
N=320					

Biology Open • Supplementary information

Table S4. ***albino*** **repair; SpRY and aNLS-SpRY, OP2* sgRNA, TÜ2270 oligonucleotide donor**

	none	weak	moderate	good	very good / complete
**SpRY**	31	17	10	11	0
	33	20	11	5	0
	113	41	34	15	0
**sum**	177	78	55	31	0
%	51.9	22.9	16.1	9.1	0
N=341					
**aNLS-SpRY**	21	17	12	20	0
	47	10	4	10	0
	95	73	88	56	0
**sum**	163	199	104	86	0
%	36.0	22.1	23.0	19.0	0
N=453					

Table S5. ***albino*** **k.o.; aNLS-Cas9 and Cas9 (IDT) 500 ng/µl**

	none	weak	moderate	good	very good / complete
**aNLS-Cas9**	6	9	23	38	123
	7	8	28	37	118
	2	6	8	25	94
	3	3	7	36	79
	0	1	4	8	69
**sum**	18	27	70	144	483
%	2.4	3.6	9.4	19.4	65.1
N=742					
**Cas9 (IDT)**	7	6	15	86	131
	7	8	24	74	131
**sum**	14	14	39	160	262
%	2.9	2.9	8.0	32.7	53.6
N=489					

Biology Open • Supplementary information
